# Improve the Colorectal Cancer Diagnosis Using Gut Microbiome Data

**DOI:** 10.3389/fmolb.2022.921945

**Published:** 2022-08-12

**Authors:** Yi-Hui Zhou, George Sun

**Affiliations:** ^1^ Department of Biological Sciences, North Carolina State University, Raleigh, NC, United States; ^2^ Binformatics Research Center, North Carolina State University, Raleigh, NC, United States; ^3^ Alston Ridge Middle School, Cary, NC, United States

**Keywords:** machine learning, prediction, feature engineering, colorectal cancer, mediation analysis, 16S rRNA

## Abstract

In the United States, colorectal cancer is the second largest cause of cancer death, and accurate early detection and identification of high-risk patients is a high priority. Although fecal screening tests are available, the close relationship between colorectal cancer and the gut microbiome has generated considerable interest. We describe a machine learning method for gut microbiome data to assist in diagnosing colorectal cancer. Our methodology integrates feature engineering, mediation analysis, statistical modeling, and network analysis into a novel unified pipeline. Simulation results illustrate the value of the method in comparison to existing methods. For predicting colorectal cancer in two real datasets, this pipeline showed an 8.7% higher prediction accuracy and 13% higher area under the receiver operator characteristic curve than other published work. Additionally, the approach highlights important colorectal cancer-related taxa for prioritization, such as high levels of *Bacteroides fragilis*, which can help elucidate disease pathology. Our algorithms and approach can be widely applied for Colorectal cancer prediction using either 16 S rRNA or shotgun metagenomics data.

## 1 Introduction

Colorectal cancer (CRC) is one of the most common and deadliest types of cancer, accounting for over 10% of all cancer deaths globally Arnold et al. (2017). The microbiome is a collection of microorganisms that reside alongside or directly on barrier surfaces, with the colony housing most of the population in mammals Gilbert et al. (2018). The colonic microbiota significantly impacts various aspects of host biology by digesting and altering nutrition and host-derived chemicals.

This study aims to find CRC at an early stage, when it is small and has not spread, often allowing for more treatment options. Some early cancers may have signs and symptoms that can be noticed, but that is not always the case. Individuals diagnosed early have a greater than 90% chance of survival Gloeckler Ries et al. (2003). More than one-third of individuals do not adhere to screening recommendations partly because of the standard diagnostics Baxter et al. (2016); colonoscopies are expensive and invasive.

Making individual-level predictions based on microbiome compositions can be very challenging. One such type of predictive modeling is to utilize microbiome compositions to predict an individual host’s phenotypes or responses, such as a health outcome or disease status, which is the most difficult. Microbiome data are very different from one sample to the next, making it hard to compare them. Many factors could change the composition of a person’s microbiome. In contrast, most of these factors or “variance components” have little or nothing to do with the response of interest. This complicated heterogeneity, combined with the high number of variables in the data, makes it very difficult to make reliable predictions about individual responses based on “noisy” compositional predictors. Making predictions with the microbiome compositions as inputs without careful pre-analysis is unlikely to succeed, no matter the prediction algorithm. Our study provides evidence that effective representation of the microbiome in terms of “features” that characterize the underlying stable signatures can enhance the predictive power. Careful generative modeling of microbiome compositions and upstream constructed factors under our control in the testbed and downstream functional profiles will identify such compositional traits.

Colorectal cancer (CRC) has many risk factors, including environmental and inherited. Fewer than 10% of patients have an indeed inherited predisposition to CRC Bogaert and Prenen (2014). There are less than 25% of patients who do not have consistently inherited syndromes, such as Peutz-Jeghers syndrome and MUTYH-associated polyposis, but with a family history of CRC. Literature also shows that many lifestyle-related factors associate with colorectal cancer Haggar and Boushey (2009). Colorectal cancer (CRC) risk factors include obesity, physical activity, smoking, alcohol intake, and certain dietary variables Khan et al. (2010). Other risk factors, such as being older, whether we have a history of adenomatous polyps (adenomas), personal history of inflammatory bowel disease, and family history of CRC or adenomas, are also risks that we cannot change Ahsan et al. (1998). This information is potentially helpful for early-stage CRC prediction. We build them into our pipeline.

In Section 2, we introduce the CRC status prediction pipeline, utilizing the risks information provided by the patients (Section 2.1). This study uses a model-based approach for feature selection (Section 2.2) instead of a fancy black box. We also use simulation to confirm the feature engineering step (Section 3). Our analysis method is rigorous, and the result has excellent potential for future patient treatment (Section 4).

## 2 Data Analysis Pipeline

**FIGURE 1:**
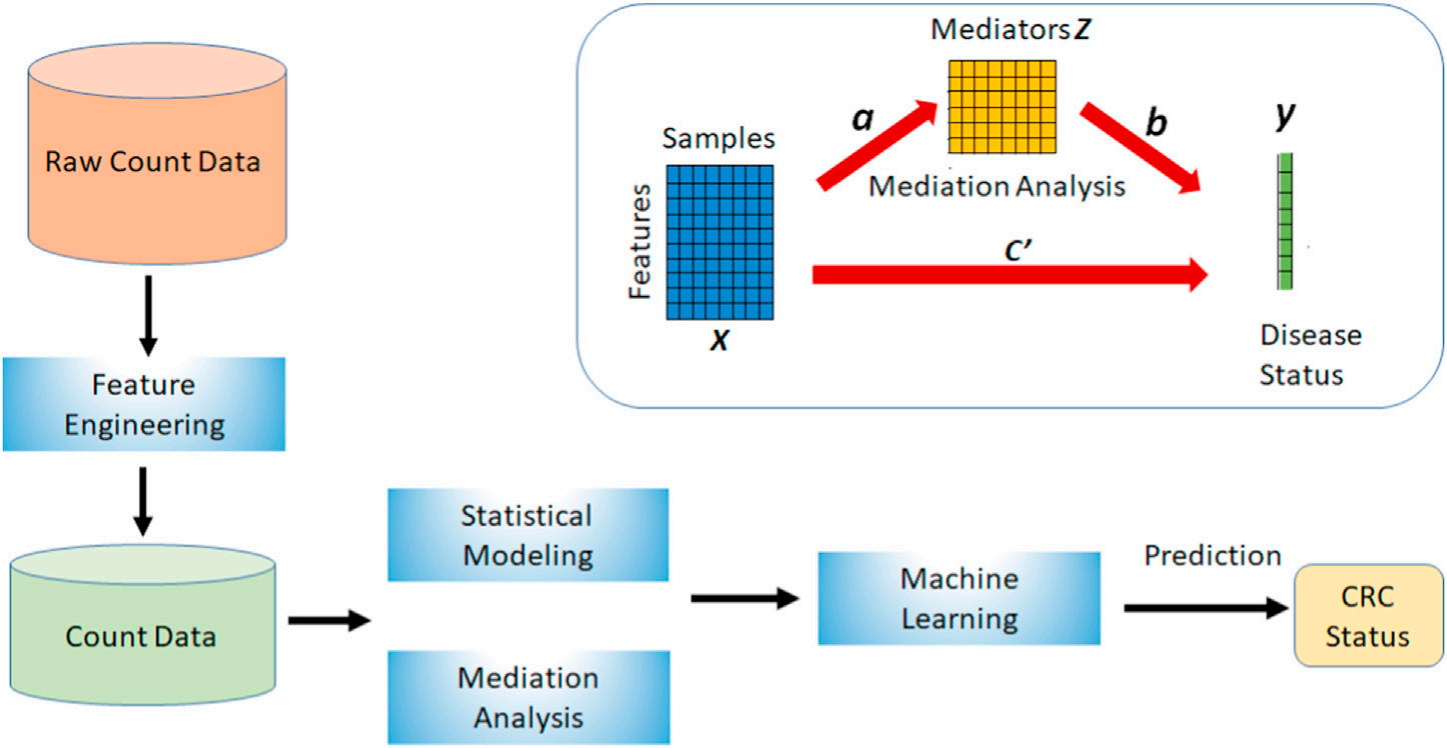
This is the Microbiome Host Trait Prediction (MHTP) pipeline to build the prediction of colorectal cancer.

**M**icrobiome **H**ost **T**rait **P**rediction (MHTP) pipeline combines the feature engineering techniques, statistical modeling of the microbiome compositions, mediation analysis, and network analysis into one framework as shown in Figure 1.

### 2.1 Feature Engineering

We provide two options for feature engineering (FE). Hierarchical feature engineering (HFE) is a feature selection method that applies phylogenetic hierarchy (i.e., an all-inclusive taxonomy) to feature engineering to classify microbiota Oudah and Henschel (2018). The algorithm combines multiple phases, which results in a set of informative features, including OTUs and taxonomy elements. Briefly, HFE uses a combination of phases: (i) an initial feature engineering phase which combines results from child taxa to synthetic “parent” taxa, (ii) a correction phase to remove redundant taxa, and (iii) a feature selection phase to keep only taxa with some minimal correlation with the outcome, and (iv) a final filtering path to remove taxa with incomplete taxonomies. We have applied it to various studies Zhou and Gallins (2019); thus, using HFE can improve the host trait prediction. However, the processed data from HFE is no longer count-data. So the output from this type of feature engineering can not align with count-based statistical modeling in the second stage of MHTP. At the end of Section 2.2, we suggest fast non-count-based statistical modeling if the user chooses HFE.

Another good choice for FE is the AUCRF approach. The area under the curve (AUC) of the random forest is calculated using all predictor variables and the answer specified in the formula argument. Then, using the specified relevance metric, we construct a ranking of predictors. We further reduce the importance of less critical variables based on their ranking. We utilize the remaining variables to calculate the AUC of a new random forest. This technique is repeated iteratively Calle et al. (2011). Additionally, we define the best set of predictive variables as the collection of variables that results in the highest out-of-bag score for the Random Forest. In the subsequent stage, we use these selected OTUs as input for statistical modeling.

### 2.2 Statistical Modeling

To further improve the colorectal cancer status’s prediction accuracy, this section introduces two statistical modeling methods to accommodate the unique microbiome data structure.

#### 2.2.1 Raw Count Data

Within each cross-validation, we apply the zero-inflated beta-binomial modeling method (ZIBB) Hu et al. (2018) to the training data. This model captures the distribution of microbiome count data and establishes a relationship with the CRC status very well. We designed ZIBB originally for discovery study, using the strategy to increase power and account for confounders by leveraging the mean-variance relationship. This statistical method has two components: a zero model for accounting for excess zeros and (ii) a count model for capturing the remaining component via beta-binomial regression, accounting for overdispersion effects. We use the OTUs highly correlated with CRC status in the testing set inside cross-validation. The selections are different in each fold.

#### 2.2.2 Relative Abundances OTU Table

In Section 2.1, using the option of HFE outputs non-count data format. In this scenario, we use the fast and robust moment-corrected correlation (MCC) algorithm Zhou and Wright (2015) in the statistical modeling stage. This approach is a fast approximation to the exact associate test of the trend that is accurate in high-throughput settings. The algorithms provide accurate *p*-values for correlation testing when we have skewed predictors and outcomes without relying on asymptotic approximation. It is faster than permutation testing but provides very similar *p*-values to permutation.

### 2.3 Mediation Analysis

Mediation analysis is a statistical procedure that employs a mediator as an intermediary in the chain connecting the independent and dependent variables. It can be used to describe the interaction between host gene expression, the gut microbiome, and clinical/health status (Carter et al., 2020). Unlike the traditional host trait prediction *Y* using microbiome count data *X* directly, causal mediation analysis aims to examine the role of a mediator or a group of mediators that lie in the pathway between an exposure and an outcome. With the expanding of ‘omics data, joint analysis of existing ‘omics data with epidemiological data through mediation analysis becomes more common (Song Y. et al., 2020). Most of the mediation methods are designed for discovery study (Wang et al., 2020).

The upper right side of Figure 1 shows a basic mediation model with a mediator variable *Z*. *X* directly influences disease status *Y* (path c’). The alternative route is the indirect route, consisting of a path from *X* to *Z* (path a) and a path from *Z* to *Y* (path b). This connection between *a* and *b* is referred to as an indirect impact or mediation effect. Both pathways illustrate the influence of *X* on *Y*. However, they are not equal because of the existence of the mediator, which has an indirect impact.

This study combines mediation analysis with our statistical modeling method to improve CRC status prediction. We apply mediation analysis for each training data and obtain the mediators that contribute additional indirect effects to the connection with CRC status. Then we stack these mediators together with the selected features and generate new training and testing data within each cross-validation. We repeat the procedure and select relevant (not necessarily the same) mediators each time.

### 2.4 Machine Learning

In the previous work, we reviewed many existing machine learning methods, including K-Nearest Neighbor, Neural Network, Elastic Net, Support Vector Machine, Lasso, Gradient Boost, Random Forest Zhou and Gallins (2019). Ensemble-of-trees methods are popular forecasting choices in regression and classification problems. Algorithm such as random forests Breiman (2001) is a well-established and widely employed procedure. Random Forest gives consistently better results than other existing popular machine learning methods in several studies regardless of the continuous or dichotomous host trait Song K. et al. (2020). Bayesian additive regression trees (BART) is a nonparametric Bayesian regression approach that uses dimensionally adaptive random basis elements. Bart’s improvements in ensemble approaches differ from predecessors in that they rely on an underlying Bayesian probability model rather than a pure algorithm. BART has shown considerable promise in a variety of simulations, and real-world applications Chipman et al. (2010). We include both Random Forest and BART in our MHTP pipeline.

## 3 Data Simulation and Result

Simulation studies are computer experiments that generate data by sampling pseudo-randomly from probability distributions with known parameters. They are essential for methodology research, especially for evaluating new approaches and comparing different methods. We assume the count data arising from 16S rRNA gene profiling or other sequencing strategy is denoted as an *m* × *n* matrix *X*_*raw*_. After feature engineering and stacking with mediators, we name the new input dataset *X*. Let $S_{j}=\sum_{i=1}^{m}$ x_ij_ be the sequencing depth of the *j*th sample. Following the zero-inflated beta-binomial (ZIBB) modeling Hu et al. (2018), we simulate data based on the cancer 1 Baxter et al. (2016) dataset. The description of this real data is in Section 4. The sample sizes for the simulated CRC cases vs controls are *n*_1_, *n*_2_ respectively. We randomly select CRC samples according to our designed structure (50, 100, respectively, for each setup). Using ZIBB modeling, the effect coefficient *β*_*i*_ for *i*th OTU is a vector with length 2, $\beta_{i}={\left(\beta_{0i},\beta_{1i}\right)}^{T}$ , where *β*_0*i*_ is the intercept. Then we use them to simulate the count matrix, given the beta-binomial distribution. We can also add the zero inflation effect by fitting the probability of zero. We remove any OTUs with complete zero counts of all samples within either group. For a randomly chosen 10% of OTUs, we specify an effect size $r=e^{\beta_{1i}}\left(1+e^{\beta_{0i}}\right)/\left(1+e^{\beta_{0i}+\beta_{1i}}\right)$ to determine *β*_1*i*_ given *β*_0*i*_. The remaining 90% of the OTUs has *β*_1*i*_’s to be zero. *D* is the design matrix with n samples and two columns. The first column of *D* were 1’s, which correspond to intercept, and the second column indicators for the two groups, of size *n*_*crc=1*_ and *n*_*crc=0*_. Using beta-binomial model, we first generate the overdispersion parameters *ψ*_*i*_ for the *i*th OTU, based on $logit\left(\psi_{i}\right)=\sum_{k=0}^{3}\gamma_{k}{mean\left(D\beta_{i}\right)}^{k}$ . We then simulate the count data using the beta-binomial distribution with all the parameters Hu et al. (2018). For each value of effect size *r*, we generate 100 datasets to test the pipeline.

**FIGURE 2:**
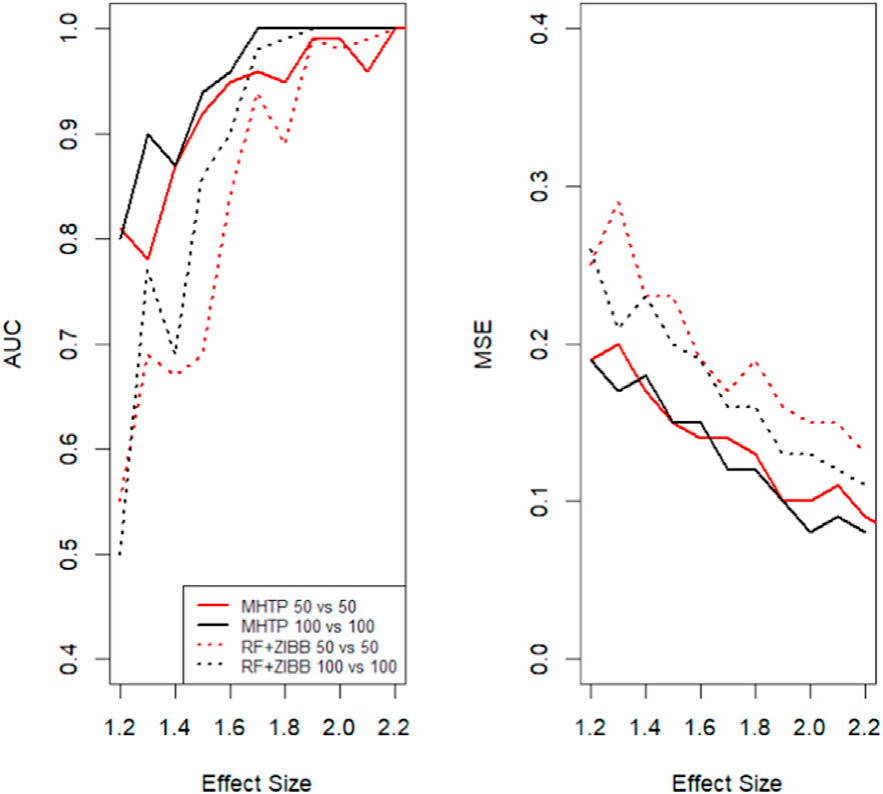
Two groups of sample sizes are used, with *n*_1_= *n*_2_=50, *n*_1_= *n*_2_=100 respectively. Here we compare two methods: 1. our MHTP approach 2. zero-inflated modeling with random forest. The left panel is the area under the curve comparison among the simulated data with the different effect sizes. The right panel shows the mean square error changes with the effect size increase.

Our MHTP pipeline consists of several processes, including feature engineering, statistical modeling, mediation analysis and the machine learning algorithm. Zhou and Gallins (2019) demonstrated that the random forest (RF) frequently makes a consistent prediction. Without loss of generality, we use RF as the final phase in this simulated scenario. Then we compare the prediction accuracy by two metrics. One is the area under the curve (AUC); Figure 2 left panel shows that the AUC increases with the increase of the effect size. An excellent model has AUC near the 1, which means it has a good separability measure. The sample size is another factor that impacts the prediction accuracy. Larger sample size data tends to have a bigger AUC, especially for the low to medium effect size. The other metric is the mean squared error (MSE), which takes the difference between the model’s predictions and the ground truth, and averages it across the whole dataset. The MSE decreases as the effect size increase, which makes perfect sense in Figure 2. We notice that MHTP outperforms other methods, particularly for low to moderate effect size/signal data. The performance of using the combination of statistical modeling and machine learning method is roughly 0.1 higher in AUC for the low effect size data, which is in between the two methods we showed in Figure 2. This pattern emphasizes the importance of feature engineering in CRC prediction.

To create a mediation relationship, we modify the method described above. First, we note that the defining feature of mediation relationships is partial or complete conditional independence of exposure/predictor data and the response values, given the mediator. Thus it is permissible to simulate *X* conditional on a mediator *z* and then *y* depending on *X* and *z*. This approach is valid even though the interpretation for applications may be reversed, i.e. the causality interpretation is *X* → *y* or *X* → *z* → *y*.

Let *Z* represent an *n* × *p* matrix of potential mediators. For our simulation purposes we will start with a latent multivariate normal *Z*′ ∼ *MVN*(0, Σ), and each variable $z_{j}=I\left[z_{j}^{\prime }>0\right]$ , i.e. the binary *z*-values are obtained by thresholding. We choose *Z*_1_ as the “true” mediator, and generate the *X* matrix following the above ZIBB simulation approach, but using *z*_1_ instead of the phenotype. Then we do a forward simulation of binary *y* values, with *P*(*Y* = 1) = expit(*α*_0_ + *α*_1_ *z*_1_ + *α*_2_ *PC*1(*X*)), which can be followed by rejection sampling if we wish to exactly control the number of sample with *y* = 0 and *y* = 1. This approach involves a mediation relationship with a single *z* and many OTUs, with effect size controlled by *α*_1_ (and *α*_1_ = 0 corresponds to no mediation and null *y*). Moreover, by including correlated columns of *Z* as specified by Σ, the identification of the “correct” mediator is not guaranteed.

Adding additional mediators helps to increase the CRC status prediction accuracy. The overall improvement of AUC is about 3% under the simulation setting with one “true” mediator. We encourage the users to include clinical information or other relevant testing results as mediators for the actual data analysis.

## 4 Real Data Analysis and Results

### 4.1 CRC Dataset 1

The first CRC dataset labeled “cancer 1” was extracted from PRJNA290926 Baxter et al. (2016). All the patients were aged above 18 years old. The V4 region of the bacterial 16 S rRNA gene was amplified using custom barcoded primers and sequenced Baxter et al. (2016). Among these 490 samples, 120 had CRC, 198 had adenomas, and 172 had no colonic lesions. We used the ones diagnosed as “cancer” and “normal” (120 vs 172). The meta data provided 25 variables, including ethnicity information, smoking status, etc.

Usually, scientists run a list of traditional machine learning methods, such as Random Forest, Xgboost, SVM, Lasso, neural network, and LDA. The area under the curve ranges from 0.53 to 0.73 for the species level with 335 OTUs. Among all these methods, Random Forest and Bart stand out. We further added feature engineering based on these two optimal methods. In this example, HFE filtered the majority of OTUs, with only eight remained, which drove the prediction less accurate. Therefore, we combine AUCRF feature engineering with count-based statistical modeling ZIBB, following our MHTP pipeline. In the last step of adding mediators, we used a Bayesian mediation method Song Y. et al. (2020) to use continuous Bayesian shrinkage priors to select mediators and assume that all the potential mediators contribute some effects in mediating the colorectal cancer status. A tiny proportion of mediators exhibit significant effects. We kept these mediators (ethnicity information, smoking status, body mass index, gender, weight) in both testing and training sets in each cross-validation step. Table 1 shows that missing any step in the MHTP pipeline will lack the power in prediction. Using our proposed method, the area under the curve is 13% higher than the optimal traditional machine learning alone, with the lowest mean square of error.

**TABLE 1:** The area under the curve, prediction accuracy, and mean square error are listed for the CRC status prediction using the real data. AUCRF refers to feature engineering in the first column; MHTP is our proposed method, which can combine with the best machine learning method.

Method	AUC	Prediction accuracy	MSE
ZIBB + Random Forest	0.777	0.732	0.191
ZIBB + Bart	0.782	0.741	0.182
AUCRF + ZIBB + Random Forest	0.804	0.757	0.168
AUCRF + ZIBB + Bart	0.802	0.743	0.180
MHTP (with RF)	0.867	0.792	0.142
MHTP (with Bart)	0.882	0.796	0.147

Normalization is an essential process to ensure the comparability of data across samples. Zhou and Gallins (2019) cited multiple works of literature, which largely account for the large variability in library sizes (total number of sequencing reads across different samples). These normalization approaches include the cumulative sum scaling, variance stabilization, trimmed-mean by M-values, and the centered log-ratio (CLR) transformation of the relative abundance vectors. There is not enough evidence that normalization has any impact on host trait prediction Song K. et al. (2020). In the analysis of CRC dataset 1, we use the internal library normalization in the step of application of statistical modeling ZIBB and also find that other normalization does not change the CRC prediction.

### 4.2 CRC Dataset 2

The second CRC dataset labeled “cancer 2” was extracted from PRJEB6070 Zeller et al. (2014). Among the collected 156 samples for the shotgun sequencing in population F, 53 patients had colorectal cancer, 42 with adenoma, and 61 were normal controls. Zeller et al. (2014) combines the 17 patients who had small adenoma with the neoplasia-free regular patients, so we followed the same 53 cases vs 88 controls here. Zeller et al. (2014) used 10-fold cross-validation and reported the area under the ROC curve 0.733 by a logistic regression model.

**FIGURE 3:**
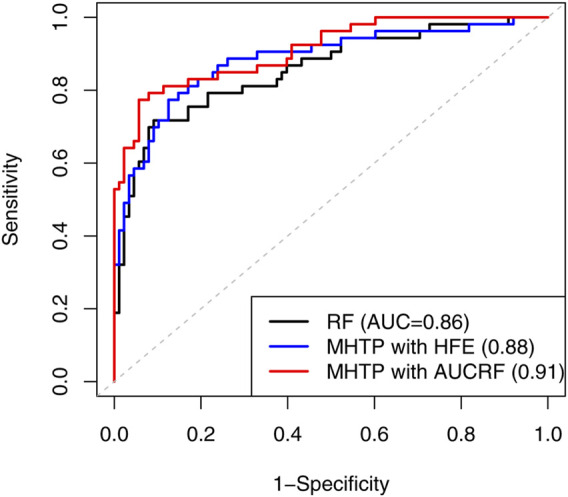
The receiver operating characteristic (ROC) curve compares the performance using Random Forest and our MHTP, on the second real CRC dataset.

We applied a list of machine learning methods like a SVM, k-nearest neighbor, neural network, Random Forests, ridge regression and Lasso. The AUC ranged from 0.51 to 0.86 under species level. The prediction accuracy decreased as we used the higher level - genus level instead. The MHTP pipeline provides two options of feature engineering procedures. HFE selected 25 OTUs, while AUCRF chose 15 out of 1,753 OTUs at the species level. The result implied that AUCRF performed better than HFE in this example. Due to the limited clinical information on cancer 2, the mediation analysis didn’t show significant mediators available, so MHTP didn’t include any clinical information for the downstream analysis. Figure 3 shows that our MHTP provides AUC 0.91, which is 0.18 higher than the logistic regression (0.73) reported by Zeller et al. (2014), indicating our pipeline provides a much better prediction accuracy, which also emphasizes the importance of feature engineering.

**FIGURE 4:**
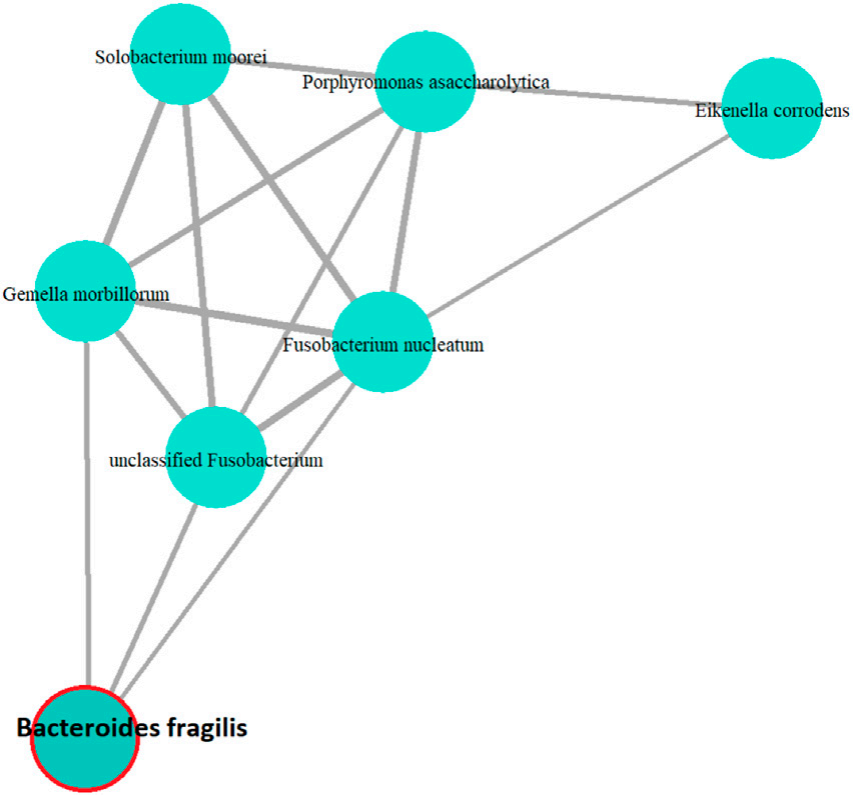
The cluster of OTUs that are highly correlated with *Bacteroides fragilis*.

*Bacteroides fragilis* is one of the top selected features. To investigate this phenomenon further, we apply the WGCNA method to identify highly co-expressed modules. The first principal component of the module containing *Bacteroides fragilis* is significantly associated with CRC status. Figure 4 shows the network graph after keeping the OTUs with pairwise correlations > 0.5. The published work Dejea et al. (2018) and Grivennikov et al. (2012) have reported that *Bacteroides fragilis* has a “pro-tumor” effect by adhering to and directly interacting with colorectal tumors, driving mutagenesis inducing inflammatory cytokines. Members of the colonic microbiome can play a detrimental role in the host’s health and potentially contribute to diseases, including colorectal cancer. The local environment can influence immune responses to intestine resident bacteria, with infection, inflammation, and nutrition all having a significant effect on the formation and differentiation of microbiome-specific T cell responses Ansaldo et al. (2019), Belkaid and Hand (2014),Wegorzewska et al. (2019). Thus, each interaction between the immune system and a microbiota member is context-dependent.

In this study, we used R packages MCC, ZIBB, randomForest (with tuning parameters ntree = 500, mtry = $\sqrt{p}$ ), and BART (with tuning parameters ntree = 50).

## 5 Conclusion

The gut microbiome plays a vital role in the host’s immune system. Our article proposed a new machine learning pipeline to boost colorectal cancer prediction accuracy, using gut microbiome and available clinical information. We integrate feature engineering, mediation analysis, statistical modeling, and an existing machine learning algorithm into a single pipeline. Moreover, we optimize the precision of colorectal cancer diagnoses, with an 8.7% higher detection rate of colorectal cancer than other published work. Our pipeline is flexible for both the 16 S rRNA microbiome data and the shotgun metagenomic data; A side product extracts and refines the taxa-taxa co-occurrence network for inferring the biological relationships between the microbes. In addition, we provide the keystone taxa related to colorectal cancer. A thorough review of the potential role of the gut and locally resident bacterial microbiota may have a beneficial impact on future cancer therapeutics.

## Data Availability

Raw fastq files are available via NCBI Sequence Read Archive (SRP062005). The two colorectal cancer datasets are PRJNA290926 and PRJEB6070.
